# Experiences of operational costs of HPV vaccine delivery strategies in Gavi-supported demonstration projects

**DOI:** 10.1371/journal.pone.0182663

**Published:** 2017-10-10

**Authors:** Siobhan Botwright, Taylor Holroyd, Shreya Nanda, Paul Bloem, Ulla K. Griffiths, Anissa Sidibe, Raymond C. W. Hutubessy

**Affiliations:** 1 Vaccine Implementation, Gavi, the Vaccine Alliance, Geneva, Switzerland; 2 Initiative for Vaccine Research, World Health Organization, Geneva, Switzerland; 3 Expanded Program on Immunization, World Health Organization, Geneva, Switzerland; 4 Health Section, UNICEF, New York, New York, United States of America; Rudjer Boskovic Institute, CROATIA

## Abstract

From 2012 to 2016, Gavi, the Vaccine Alliance, provided support for countries to conduct small-scale demonstration projects for the introduction of the human papillomavirus vaccine, with the aim of determining which human papillomavirus vaccine delivery strategies might be effective and sustainable upon national scale-up. This study reports on the operational costs and cost determinants of different vaccination delivery strategies within these projects across twelve countries using a standardized micro-costing tool. The World Health Organization Cervical Cancer Prevention and Control Costing Tool was used to collect costing data, which were then aggregated and analyzed to assess the costs and cost determinants of vaccination. Across the one-year demonstration projects, the average economic and financial costs per dose amounted to US$19.98 (standard deviation ±12.5) and US$8.74 (standard deviation ±5.8), respectively. The greatest activities representing the greatest share of financial costs were social mobilization at approximately 30% (range, 6–67%) and service delivery at about 25% (range, 3–46%). Districts implemented varying combinations of school-based, facility-based, or outreach delivery strategies and experienced wide variation in vaccine coverage, drop-out rates, and service delivery costs, including transportation costs and per diems. Size of target population, number of students per school, and average length of time to reach an outreach post influenced cost per dose. Although the operational costs from demonstration projects are much higher than those of other routine vaccine immunization programs, findings from our analysis suggest that HPV vaccination operational costs will decrease substantially for national introduction. Vaccination costs may be decreased further by annual vaccination, high initial investment in social mobilization, or introducing/strengthening school health programs. Our analysis shows that drivers of cost are dependent on country and district characteristics. We therefore recommend that countries carry out detailed planning at the national and district levels to define a sustainable strategy for national HPV vaccine roll-out, in order to achieve the optimal balance between coverage and cost.

## 1. Introduction

Cervical cancer, predominantly caused by infection with human papillomavirus (HPV), is the fourth most common cancer in women worldwide [1–3]. Over 85% of the global burden of cervical cancer occurs in low- and middle-income countries (LMICs), where nationwide cervical cancer screening and treatment is limited [1,4]. Two safe and efficacious vaccines, available in bivalent and quadrivalent forms, protect against 70% of cancer-causing HPV infections and have been pre-qualified by the World Health Organization (WHO) [5]. HPV vaccination is a highly cost-effective intervention for cervical cancer control in LMICs [6,7]. As part of the Global Vaccine Action Plan (GVAP), WHO has recognized the severity of the worldwide burden of HPV and cervical cancer and is recommending introduction of HPV vaccine for adolescent girls into national immunization programs [8]. WHO currently recommends two doses of the HPV vaccine for girls aged nine to fourteen, having altered its recommendation from three to two doses in 2014 [9,10].

Cost is a potential barrier for countries that are considering introducing HPV vaccine into national immunization programs, as like other new vaccines, it remains considerably more expensive than traditional vaccines. Gavi, the Vaccine Alliance, has accelerated access to HPV vaccines in low-income countries by negotiating vaccine prices with manufacturers, securing the bivalent vaccine at US$4.50 and the quadrivalent vaccine at US$4.60 per dose [11,12]. Furthermore, since many countries may have limited experience in reaching adolescent girls for vaccination, Gavi supported demonstration projects between 2012 and 2016, enabling countries to assess the coverage, feasibility, acceptability, and cost of their vaccination strategy before continuing to national scale-up [11]. During the two-year demonstration projects, Gavi provided each country with sufficient HPV vaccine supply to vaccinate a target population of up to 15,000 adolescent girls and cash support for operational costs (excluding vaccine). The cash support totalled either US$4.80 per girl or $50,000 in the first year, whichever was greater, and either US$ 2.40 per girl or $25,000 during the second year [5,9].

Although demonstration projects have indicated that HPV vaccination is feasible in LMICs, there remain concerns around operational costs, especially as there is no established delivery system to reach adolescents in many low-income countries [12–14]. Adolescent girls are not normally targeted by routine immunization programs outside of mass single-dose or catch-up campaigns targeting multiple age cohorts, such as measles and rubella vaccination, and delivery of two doses to adolescent girls can challenge existing health systems, resulting in high costs. This study aimed to determine the costs of HPV vaccine delivered during Gavi demonstration projects in twelve countries, to ascertain the cost distribution of different activities, and to analyze the cost implications and drivers of different vaccine delivery strategies. These findings will inform country-level policymakers and planners, WHO, Gavi, and other stakeholders regarding the first experiences of HPV vaccine introduction in LIMCs and assist in decision-making for national and pilot vaccination programs.

## 2. Data, materials and methods

### 2.1 WHO C4P costing tool

The WHO Cervical Cancer Prevention and Control Costing (C4P) tool has been developed to support LMICs without prior experience of scaling up comprehensive programs for cervical cancer prevention and control for planning and costing alternative strategies. The tool is built in MS Excel 2010 and consists of two modules: i) HPV vaccination, and ii) cervical cancer screening and treatment [15]. This analysis focused exclusively on the HPV vaccination module of the C4P tool. The C4P tool considers both financial cost, which consists of new expenditure for the intervention, and economic cost, which also includes resources that are already in place and donations [16]. The HPV vaccination module consists of eight cost components (Table 1) and focuses on the incremental costs of HPV vaccination on top of existing immunization services [16]. The C4P tool differentiates between investment costs, which are capital costs required at the beginning of the program, and recurrent costs, which include operational costs of the program like vaccine transport and health worker salaries [16]. C4P has been used at two stages by countries carrying out small-scale Gavi demonstration projects: during the first year or two years of the project to retrospectively estimate the cost of demonstration programs, and in subsequent years to prospectively model future costs of nationwide scale-up.

**Table 1:** Summary of cost components included in the C4P tool.

	Cost component	Resources
**Investment costs**	**Microplanning ^**a**^**	• Per diems and travel allowances • Venue rental • Transport • Personnel time spent in meetings ^b^
	**Training ^**a**^**	• Development and production of training materials • Per diems and travel allowances • Venue rental • Transport • Stationery • Personnel time spent in training ^b^
	**Social mobilization and IEC ^**a**^**	• Facilitator time in meetings • Per diems and travel allowances • Stationery • Production of TV/radios pots, posters, leaflets • Value of personnel, teacher, and volunteer time ^b^
	**Cold chain supplement**	• Cold chain equipment (annualized) (annualized and discounted) ^b^
	**Other**	• Purchase of incinerators for waste disposal (annualized) (annualized and discounted) ^b^
**Recurrent costs**	**Vaccines**	• Vaccines and injection supplies (cost to government) • Freight, clearance, insurance, taxes • Vaccines and injection supplies (cost to donors) ^b^
	**Service delivery**	• Transport fuel and maintenance • Per diems and travel allowances • Supplies (e.g. cotton) • Value of personnel time spent on vaccination ^b^
	**Monitoring and evaluation**	• Travel allowances • Transport fuel and maintenance • Stationery • Tally sheets and registers • Vaccination cards • Surveillance materials • Value of personnel time ^b^
	**Other**	• Transport and fuel for waste management

C4P, WHO Cervical Cancer Prevention and Control Costing Tool. IEC, information, education, and communication.

a Introduction cost

b Costs included only in economic cost estimates (not financial)

### 2.2. Data

This analysis uses cross-sectional retrospective cost estimates generated by the C4P tool for Gavi demonstration projects. The dataset includes twelve countries that carried out C4P HPV costing studies and submitted them to Gavi between January 2013 and August 2016: Cameroon, the Gambia, Ghana, Kenya, Lao People’s Democratic Republic (PDR), Madagascar, Malawi, Niger, Senegal, Solomon Islands, the United Republic of Tanzania, and Zimbabwe. As the C4P tool for Zimbabwe was completed using a modified version of the C4P Tool, this country was excluded from the dataset for regression analysis. In most countries, costs were only estimated in the first year of the demonstration projects, but in two countries costs were also estimated in the second year after altering the delivery strategy. With the exception of one country that vaccinated five cohorts, all of the demonstration projects targeted a single-year birth cohort or a single school grade of girls. The Gavi demonstration countries delivered either two or three doses of HPV vaccine; this variation is due to the change in the WHO recommendation in 2014 during the period of data collection [9,10]. The countries employed varying combinations of school-based, health facility-based, or outreach-based delivery strategies. For each country, the tool was completed with technical assistance from PATH, WHO, CDC, personnel from the national government, or experts from academia or local non-governmental organizations (NGOs). For the regression analysis, additional data were collected from The World Bank and WHO, including gross national income (GNI), urban population proportions, diphtheria-tetanus-pertussis third vaccine dose (DTP3) coverage, gross domestic product (GDP) deflation rate, and purchasing power parity (PPP) conversion factors. DTP3 was used as a proxy for the strength of the immunization system.

### 2.3 Analysis

Data from each country were entered into the most recent version of the C4P demonstration tool (DEMOv1.6.1) and converted to 2013 US dollar values using inflation rates from the World Bank. Information was then extracted from each completed tool and entered into a Microsoft Excel database (S1 Table). In the case of missing or incomplete data, data points were either extracted from WHO country-specific comprehensive multi-year plans (cMYPs) [17] or determined in consultation with the economist who completed the C4P tool for a country. For this analysis, coverage was defined as second-dose coverage, drop-out as the difference between the first and second dose, and cost per dose was considered instead of cost per fully immunized girl (FIG). Statistical analysis was carried out in Python version 3.4.3 and Stata version 14.

## 3. Results

### 3.1 Description of the dataset

#### 3.1.1 Country & district characteristics

Of the twelve countries included in this analysis, five are located in West Africa, five in East Africa, one in Southeast Asia, and one in the Western Pacific (Table 2). Six countries delivered a three-dose schedule of the HPV vaccine and six countries delivered a two-dose schedule (Table 2). Five countries used the bivalent HPV vaccine and seven countries used the quadrivalent vaccine. The country target population size ranged from 5,274 to 22,635 girls, with an average size of 7,495. Average GNI per capita was US$2,240, ranging from US$780 to US$4,550. Countries with higher GNI per capita tended to have higher school enrolment, but there was no relationship between GNI per capita and DTP3 coverage or target population size. A total of 23 districts were included in the analysis.

**Table 2:** Baseline characteristics of demonstration project countries.

Region, no. (%)	Statistic at baseline
• West Africa	5 (42)
• East Africa	5 (42)
• Southeast Asia	1 (8)
• Western Pacific	1 (8)
HPV vaccine type, no. (%)	
• Bivalent	5 (42)
• Quadrivalent	7 (58)
HPV vaccine dose schedule, no. (%)	
• 2 doses	6 (50)
• 3 doses	6 (50)
GNI (2014 US$), median (IQR)	2240 (1528–2730)
Urban population (%), median (IQR)	33 (24–46)
Target population size, median (IQR)	7495 (6168–15402)
DTP3 vaccine coverage (%), median (IQR)	89 (84–96)
School enrolment (%), median (IQR)	95 (85–98)

HPV, human papillomavirus. IQR, inter-quartile range. GNI, gross national income. DTP3, diphtheria-tetanus-pertussis. US$, United States dollars.

#### 3.1.2 Demonstration project design

Eleven countries selected two districts for the demonstration project and one country selected one district, with seven countries selecting one urban and one rural district. All countries included school-based delivery, but only one country carried out vaccination through an existing school health program while others used campaign delivery. To reach out-of-school girls, five countries additionally included health facility-based delivery and four delivered through outreach. Across these five countries, between 82% and 100% of doses were administered through schools. Four countries also delivered HPV vaccine by outreach.

#### 3.1.3 Vaccine coverage

Districts achieved coverage between 64% and 99%, with mean coverage of 83% (Table 3). In general, the highest coverage was achieved with school-based delivery at 85% (±12.5%), and the lowest coverage occurred through health facility-based delivery at 50% (±31.8%). Since the vaccine was predominantly delivered through schools, high school-based coverage was indicative of high overall coverage. The drop-out rate between the first and second doses ranged from 0% to 19%. Drop-out rates between the first and second dose were 7% for school, 11% for facility, and 11% for outreach delivery strategies. While not all countries administered a third dose, drop-out rates between second and third doses were comparable to those between first and second doses.

**Table 3:** HPV vaccination coverage and drop-out rates of demonstration project districts.

HPV vaccination coverage and drop-out rates	Mean rate (%), SD
Doses administered through school-based delivery	93 (13.2)
Second-dose coverage	83 (12.8)
Second-dose coverage by delivery strategy	
• School	85 (12.5)
• Health facility	50 (31.8)
• Outreach	62 (34.4)
Drop-out rate between first and second doses	6 (6.0)
Drop-out rate between first and second doses by delivery strategy	
• School	7 (5.7)
• Health facility	11 (7.5)
• Outreach	11 (10.3)

HPV, human papillomavirus. SD, standard deviation.

### 3.2 Program costs

#### 3.2.1 Average costs in year one

The mean economic cost per dose for all demonstration projects was US$19.98 (range US$10.93 –US$56.60) and the mean financial cost per dose was US$8.74 (range US$3.39 –US$25.61) (Table 4). On average, introduction costs comprised 46% of total financial costs (range 15%– 77%), and comprised 32% of economic costs (range 10%– 58%). Economic costs were approximately two and a half times higher than financial costs.

**Table 4:** HPV vaccine economic and financial costs of demonstration project countries, 2014 US$.

Dose schedule	Financial cost		Economic cost	
	Mean cost per dose	SD	Mean cost per dose	SD
**Overall (N = 12)**	8.74	5.8	19.98	12.5
**Two-dose schedule (N = 6)**	10.48	7.5	23.75	16.2
**Three-dose schedule (N = 6)**	6.83	2.2	15.87	4.7

HPV, human papillomavirus. US$, United States dollars. SD, standard deviation.

#### 3.2.2 Cost components in year one

The greatest contributor to economic costs was vaccine procurement, which represented approximately half of the total economic cost per dose in nearly all Gavi demonstration projects (Table 5). The vaccination economic cost component had a range of US$5.07 –US$8.58, comprising 29% to 57% of the total economic cost per dose. Social mobilization/information, education, and communication (IEC) and service delivery each comprised about 20% of economic cost.

**Table 5:** Breakdown of economic and financial cost per HPV vaccine dose in demonstration project countries, 2014 US$.

Cost component	Financial cost		Economic cost	
	Mean cost per dose	SD	Mean cost per dose	SD
**Microplanning**	0.50	0.58	1.45	1.65
**Vaccine**	0.44	0.27	6.70	1.07
**Training**	1.19	1.61	1.69	2.23
**Social mobilisation and IEC**	2.75	2.13	3.56	3.43
**Service delivery**	1.84	1.44	3.31	3.31
**Monitoring and evaluation**	0.98	0.99	1.83	3.06
**Cold chain**	0.01	0.03	0.01	0.03
**Other**	0.53	0.78	0.68	0.88
**TOTAL**	8.74	5.8	19.98	12.5

HPV, human papillomavirus. IEC, information, education, communication.

Social mobilization/IEC and service delivery comprised the greatest proportion of financial costs, at around 30% and 25% respectively, although there was wide variation be-tween countries (Table 5). The next greatest shares of the financial cost per dose were comprised of training and monitoring & evaluation at approximately 10% each. Social mobilization was a key contributor to financial cost, and countries incurring the greatest financial costs also had the highest social mobilization/IEC costs.

#### 3.2.3 Service delivery costs by delivery strategy

School-based delivery had the highest service delivery cost per dose, with an average financial cost of US$1.80 per dose and an average economic cost of US$3.33 per dose (Table 6). Facility-based delivery featured the least costly service delivery, with US$0.07 financial cost per dose and US$0.09 economic cost per dose. Outreach-based delivery had an average financial cost of US$0.27 per dose and an average economic cost of US$0.57 per dose. School and outreach incurred costs for transportation and health worker per diems, which health facility based delivery did not (aside from one country, which paid per diems to health workers for facility-based delivery). Outreach costs were lower than school costs because a greater number of girls were vaccinated per visit on average. In general, the highest second-dose coverage was achieved with school-based delivery at 85%, and the lowest coverage occurred through health facility-based delivery at 50%.

**Table 6:** Economic and financial costs per dose of service delivery cost component (2014 US$).

Service delivery cost component by strategy	Mean cost in 2014 US$ (SD)
Economic cost per dose of service delivery by strategy	
• School-based	3.33 (3.3)
• Health facility-based	0.09 (0.3)
• Outreach-based	0.57 (0.8)
Financial cost per dose of service delivery by strategy	
• School-based	1.80 (1.5)
• Health facility-based	0.07 (0.3)
• Outreach-based	0.27 (0.4)

US$, United States dollars. SD, standard deviation.

#### 3.2.4 Cost profile in year two

In the two countries that estimated costs for the second year, year two costs were lower than those of year one, primarily because introduction costs substantially decreased. In one of the countries, mebendazole for deworming of girls and boys aged nine to thirteen was integrated with HPV vaccination visits. Overall cost per dose in the first country was 25% lower in year 2, mostly from lower investment in social mobilization/IEC, which did not adversely impact coverage. Training and microplanning activities continued for integrated delivery, but the deworming tablets themselves contributed to less than 3% of total year two costs.

The second country moved from school-based campaigns in year one and instead incorporated HPV delivery with the routine vaccination system in year two. Financial costs decreased by 70% as no introductory activities except microplanning were carried out and there was no budget for the service delivery cost component, as this was instead absorbed by the routine vaccine delivery system.

### 3.3 Factors influencing cost

#### 3.3.1 Relationship between cost and coverage

Regression analysis indicated that there was no statistically significant relationship between economic or financial cost and coverage, or between social mobilization and coverage. No cost component showed a statistically significant relationship with coverage.

#### 3.3.2 Cost determinants

**Fig 1:**
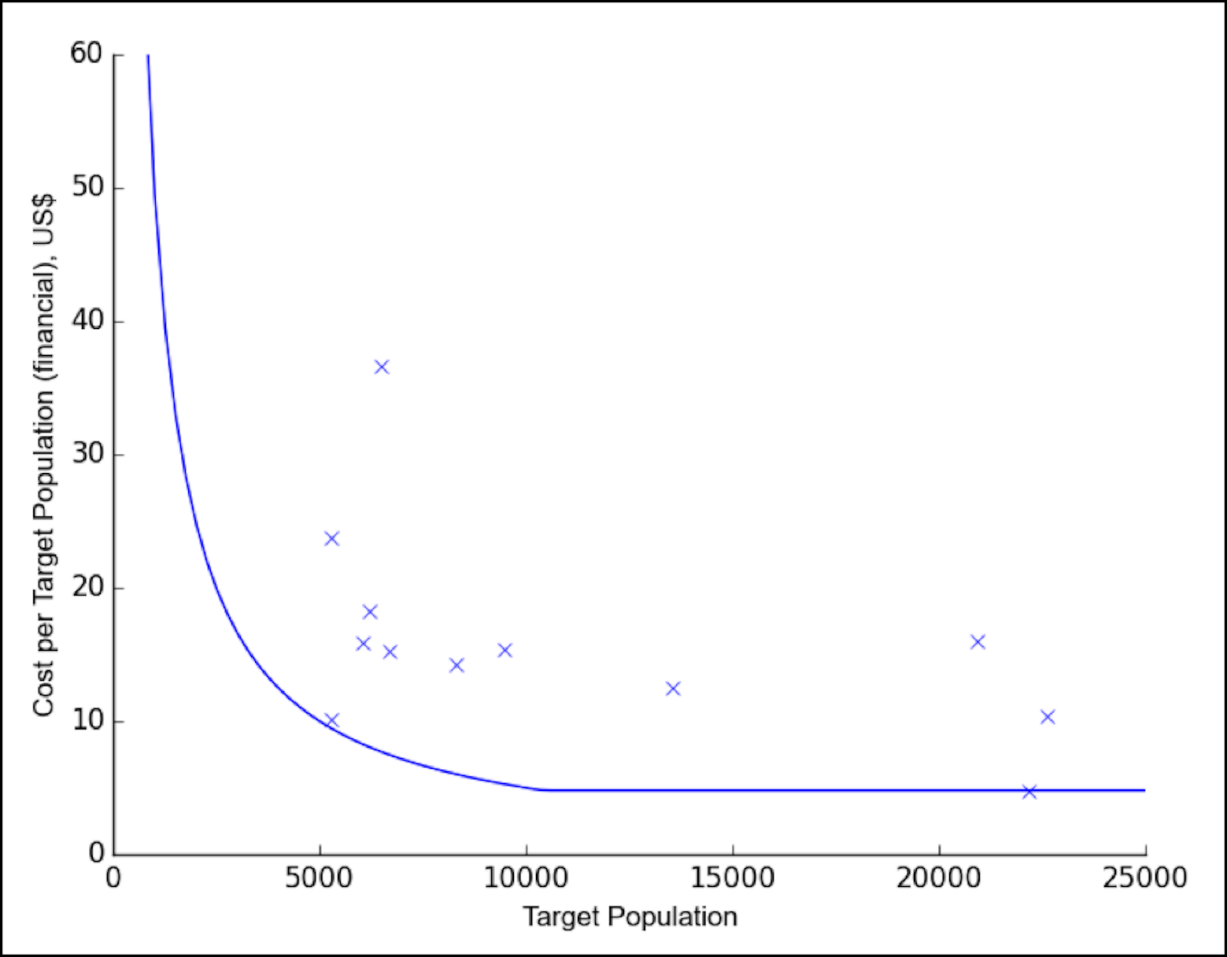
Target population and financial cost per dose in demonstration project countries. US$, United States dollars. Blue curve represents theoretical cost per girl in the target population if countries were to spend exactly the amount provided by the Gavi demonstration project first year grant. The points (denoted by “x”) represent the actual financial cost per girl in the target population from Gavi demonstration projects.

Countries with a smaller target population in the demonstration project had higher financial cost per dose, particularly when the target group was fewer than 12,000 girls (Fig 1). Regression analysis between financial cost per dose and target population showed a significant relationship in regression analysis (R = 0.4447, p = 0.025). Costs in Zimbabwe, excluded from regression analysis, were also driven by target population: service delivery costs were reduced by half by delivering the second vaccine dose for the first cohort of girls concurrently with the first vaccine dose for the second cohort. Contrary to previous studies [18–21], health worker salary was not found to be a significant cost driver of service delivery in the regression analysis.

#### 3.3.3 Country and district characteristics

Financial cost per dose decreases as the number of girls vaccinated increases. In all countries, districts with fewer schools per target population–indicative of fewer school visits–incurred lower financial school delivery costs per dose, regardless of variations in per diem, salary and transport costs. Average length of time to reach an outreach post influenced cost for outreach service delivery. Similarly, school based delivery is less expensive either when schools are within a short distance of health facilities, or when there is a high density of schools within an area. As length of time to reach a school increases, health worker per diem becomes a greater driver of cost.

## 4. Discussion

### 4.1 Demonstration project costs and transition to national introduction

Our analysis found an average economic cost per dose of US$19.98 and an average financial cost per dose of US$8.74. This is within the range of operational costs found in non-Gavi pilot projects [14], but is still significantly higher than the delivery costs of routine EPI vaccines [13, 20–23]. By contrast, the financial cost per FIG to deliver a three-dose schedule in countries with national HPV vaccination programs was much lower: $10.23 in Rwanda and $7.58 in Bhutan with school-based campaign strategies [16,19,24]. Accordingly, for several reasons it is anticipated that cost per dose in Gavi countries will decrease as countries transition to national roll-out.

Firstly, demonstration projects are resource-intensive, requiring coordination and mobilization from the national level for vaccination of a relatively small number of girls. With national introduction, countries should be able to take advantage of economies of scale. Secondly, due to Gavi funding for operational costs in demonstration projects and a prerequisite to reach 50% coverage [11], countries may not have prioritized the most optimal and efficient strategy compared to a situation with no external support. The curve superimposed on Fig 1 shows the Gavi cash support provided per girl: financial cost per target girl follows a similar pattern to Gavi cash support per target girl, suggesting Gavi cash support may have influenced spending. For national introduction, restricted budgets will require countries to place a greater focus on efficiency and limit spending. Thirdly, the demonstration projects in this analysis were seldom integrated within routine national immunization infrastructure, and were instead delivered by a campaign-like approach, which may have inflated costs. Rwanda has shown that cost per dose can significantly decrease by leveraging existing routine outreach systems to deliver HPV vaccine to schools, as opposed to delivering the vaccine through campaigns. It should however be noted that Rwanda’s success in sustaining high vaccination coverage has been attributed to strong social mobilization during the first few years of HPV vaccine introduction [19,25], suggesting that integration with routine systems may still require intensive campaign-style social mobilization to achieve high coverage. Fourthly, as a new vaccine targeting an underserved population, HPV vaccination programs incur high introduction costs, primarily due to intensive social mobilization, communication, and microplanning activities. Results from demonstration programs suggest that national costs could decrease by a factor of two within the first few years of HPV introduction, once introduction activities cease [16,24]. Finally, countries will benefit from collective lessons learned from demonstration projects, allowing them to make strategic decisions for national introduction that balance cost and coverage.

### 4.2 Key learnings to inform HPV vaccination programs

#### 4.2.1 Planning at the national and district level is required to determine the optimal delivery strategy

On the surface, our analysis appears to show that school-based delivery achieved highest coverage at the highest cost, whereas facility-based delivery achieved the lowest coverage at lower cost. However, there are a number of limitations to this interpretation. All countries in the dataset delivered by campaign to school–typically characterized by per diems, transportation costs, and intensive supervision–which factors into the higher school delivery costs, whereas it is possible to deliver to school through established routine outreach activities or integrated with existing well-functioning school health programs, which would be less costly. Similarly, health facility-based costs may be higher in settings where per diems are given to health workers or when extra health workers are employed by the National Immunization Program (NIP) for HPV vaccine introduction. Finally, our analysis may underestimate coverage for facility and outreach delivery, since countries in the dataset only used these strategies to reach out-of-school girls, a hard-to-reach population.

Our analysis suggests that countries should take a pragmatic, context-based approach in defining their delivery strategy. This suggestion is supported by data from Bhutan, where a mixed delivery strategy in the third year, dependent on district characteristics, managed to boost coverage to above 90% with lower costs than school based delivery [24]. For example, in areas with few eligible girls registered per school or high rates of absenteeism, it may be more economical to deliver HPV vaccine from a central outreach post and mobilize girls from multiple surrounding schools to access the vaccination point. Similarly, if outreach posts or schools are far from health facilities, it may be worth considering facility based delivery or leveraging existing outreach activities, such as deworming. If school-based delivery is seen as the only viable option to reach high coverage in certain settings, but costs are prohibitive, countries can exploit economies of scale to reduce costs by carrying out annual vaccination, in which the vaccine is delivered on a twelve-month schedule, such that the second dose for one cohort of girls is delivered concurrently with the first dose for the next cohort. Alternatively, such countries could use the Gavi vaccine introduction grant to make a large investment in social mobilization and introduce the vaccine via school delivery and outreach campaigns, but later transition to a more affordable facility-based model after achieving high HPV vaccine awareness. It is important to note that countries without existing infrastructure for school-based delivery will incur higher costs for school delivery. Countries should take a long-term view to invest in school health programs to benefit a range of health interventions for school children, including HPV vaccination.

#### 4.2.2 Evidence-based social mobilization is required to boost coverage and decrease cost

HPV vaccination requires greater social mobilization than traditional vaccines because of sensitivity surrounding sexual activity in certain settings, the gender-specific target population, and the lack of natural demand from low cervical cancer awareness in Gavi countries. However, in this analysis social mobilization spending did not affect coverage but did influence overall cost, suggesting that social mobilization funds were not optimally spent. It is imperative that countries design evidence-based social mobilization strategies, based on existing literature [26,27, 28].

#### 4.2.3 Sustainability of HPV vaccination programs

Vaccine cost represents around half of the economic cost of HPV vaccination, which has implications for program sustainability. This is also true for childhood vaccines, as the cost of vaccine procurement and vaccine delivery are almost equal [13].A multi-country study found that many countries perceive the financial cost of vaccine delivery as being very high [26], suggesting a limited capacity to shoulder HPV vaccination costs.

However, the HPV vaccine only targets half of each age cohort, thus the resources required for HPV vaccine introduction may actually have a smaller impact on a country’s fiscal space than other new vaccines when administered in a routine, sustainable manner. Budget impact analyses are needed to further demonstrate the impact of HPV vaccination on country fiscal space.

### 4.3 Limitations

The primary limitation in this study is the size and validity of the dataset. The dataset is small, dominated by campaign school-based delivery strategies, and includes districts that may not be representative of their country context. For example, districts in Gavi demonstration projects were frequently chosen due to high school enrolment, strong vaccination program performance, or ease of access. Furthermore, data quality varied between countries and information may be subject to recall bias due to the nature of retrospective cost estimates.

Another limitation comes from the C4P tool itself. The tool only disaggregates costs by delivery strategy in the service delivery cost component (Table 1), preventing analysis of differences in social mobilization, training, supervision, or microplanning costs. Countries are therefore encouraged to build different costing scenarios for national roll-out to provide a more holistic view of delivery strategy options. Finally, the C4P tool cannot capture certain factors that influence coverage and cost, such as strong leadership and engagement of stakeholders across health, finance, and education [26]. However, despite these limitations, our analysis provides important considerations for countries introducing the HPV vaccine, either as a pilot or at national scale.

## 5. Conclusion

To the best of our knowledge this is the first study that describes the operational costs of HPV vaccination in small-scale demonstration projects across low-income countries using a standardized micro-costing tool. Although the costs per dose from our analysis appear high (US$8.74 financial and US$19.98 economic), costs are expected to decrease substantially with national introduction. Furthermore, findings from this cost analysis should equip countries to define the optimal and cost-effective country-specific strategies.

Our analysis identified social mobilization and service delivery as the main drivers of financial costs, and vaccine cost as the main economic cost driver. Service delivery costs increase for school-based delivery if few girls are vaccinated per school or if health workers have to travel a long distance to reach the outreach post or school.

The optimal strategy for large-scale implementation will depend on country and district-level characteristics. We recommend that countries utilize the Gavi Vaccine Introduction Grant (VIG) to carry out detailed planning at the district level, together with a costing exercise of the different options, to define a strategy for HPV vaccine roll-out that achieves the optimal balance between coverage and cost.

Moving forward, as LMICs begin to roll-out HPV vaccine at a national scale, it is important to continue to collect costing data and to complete an analysis of national cost data to further inform future vaccination strategies.

## Supporting information

S1 Table Parameters included in C4P country database. C4P, Cervical Cancer Prevention and Control Costing Tool. GNI, gross national income. PPP, purchasing power parity. i$, international dollars. DTP3, diphtheria-tetanus-pertussis third-dose. US$, United States dollars. FIG, fully immunized girl.
(DOCX)
