# Supplementary material for: Experiences of operational costs of HPV vaccine delivery strategies in Gavi-supported demonstration projects
Source: PLoS One. 2017 Oct 10;12(10):e0182663. doi: 10.1371/journal.pone.0182663 (PMC5634534; doi:10.1371/journal.pone.0182663)
Supplement: S1 Table — C4P, Cervical Cancer Prevention and Control Costing Tool. GNI, gross national income. PPP, purchasing power parity. i$, international dollars. DTP3, diphtheria-tetanus-pertussis third-dose. US$, United States dollars. FIG, fully immunized girl. (DOCX) [file pone.0182663.s001.docx]

**S1 Table. Parameters included in C4P country database.**

| Category | Parameters |
| --- | --- |
| Country characteristics | Region, GNI per capita (PPP, i$), Terrain (flat or mountainous), Population in urban areas, Target population, DTP3 coverage, School enrolment |
| Vaccine administration | Dose schedule, Vaccine type |
| Operational costs | Total economic costs (US$), Total financial costs (US$), Economic cost per dose, Financial cost per dose, Economic cost per dose by cost component, Financial cost per dose by cost component, Economic cost per FIG, Financial cost per FIG, Economic cost per dose of service delivery by delivery strategy, Financial cost per dose of service delivery by delivery strategy |
| Coverage and drop-out | First-dose coverage, second-dose coverage, third-dose coverage, drop-out rate between first and second doses, drop-out rate between second and third doses, Dose coverage by delivery strategy, Drop-out rate by delivery strategy |
| Immunization activities | Number of microplanning activities, Number of social mobilization activities, Number of vaccinators trained, Number of supervision visits, Time spent on vaccination, Number of schools per target population, Number of vaccinators per target population, Health worker salary and per diem, Transport costs, Total number of doses administered |

C4P, Cervical Cancer Prevention and Control Costing Tool. GNI, gross national income. PPP, purchasing power parity. i$, international dollars. DTP3, diphtheria-tetanus-pertussis third-dose. US$, United States dollars. FIG, fully immunized girl.
